# Lung Cancer Screening in Brazil Comparing the 2013 and 2021 USPSTF Guidelines

**DOI:** 10.1001/jamanetworkopen.2023.46994

**Published:** 2023-12-11

**Authors:** Isabel Cristina Martins Emmerick, Mônica Rodrigues Campos, Debora Castanheira, Jessica Muzy, Aline Marques, Luisa Arueira Chaves, Mario Jorge Sobreira da Silva

**Affiliations:** 1 Division of Thoracic Surgery, Department of Surgery, UMass Chan Medical School, Worcester, Massachusetts; 2 Departamento de Ciências Sociais, Escola Nacional de Saúde Pública, Fundação Oswaldo Cruz, Rio de Janeiro, Brazil; 3 Laboratório de Pesquisa Clínica em DST e Aids, Instituto Nacional de Infectologia Evandro Chagas, Fundação Oswaldo Cruz, Rio de Janeiro, Brazil; 4 Laboratório de Informações em Saúde, Instituto de Comunicação Científica e Tecnológica em Saúde, Fundação Oswaldo Cruz, Rio de Janeiro, Brazil; 5 Instituto de Ciências Farmacêuticas, Universidade Federal do Rio de Janeiro, Macaé, Rio de Janeiro, Brazil; 6 Instituto Nacional de Câncer, Coordenação de Ensino, Rio de Janeiro, Brazil

## Abstract

**Importance:**

It is estimated that, from 2023 to 2025, lung cancer (LC) will be the second most frequent cancer in Brazil, but the country does not have an LC screening (LCS) policy.

**Objective:**

To compare the number of individuals eligible for screening, 5-year preventable LC deaths, and years of life gained (YLG) if LC death is averted by LCS, considering 3 eligibility strategies by sociodemographic characteristics.

**Design, Setting, and Participants:**

This comparative effectiveness research study assessed 3 LCS criteria by applying a modified version of the LC-Death Risk Assessment Tool (LCDRAT) and the LC-Risk Assessment Tool (LCRAT). Data are from the 2019 Brazilian National Household Survey. Participants included ever-smokers aged 50 to 80 years. Data analysis was performed from February to May 2023.

**Exposures:**

Exposures included ever-smokers aged 50 to 80 years, US Preventive Services Task Force (USPSTF) 2013 guidelines (ever-smokers aged 55 to 80 years with ≥30 pack-years and <15 years since cessation), and USPSTF 2021 guidelines (ever-smokers aged 50 to 80 years with 20 pack-years and <15 years since cessation).

**Main Outcomes and Measures:**

The primary outcomes were the numbers of individuals eligible for LCS, the 5-year preventable deaths attributable to LC, and the number of YLGs if death due to LC was averted by LCS.

**Results:**

In Brazil, the eligible population for LCS was 27 280 920 ever-smokers aged 50 to 80 years (13 387 552 female [49.1%]; 13 249 531 [48.6%] aged 50-60 years; 394 994 Asian or Indigenous [1.4%]; 3 111 676 Black [11.4%]; 10 942 640 *Pardo* [40.1%]; 12 830 904 White [47.0%]; 12 428 536 [45.6%] with an incomplete middle school education; and 12 860 132 [47.1%] living in the Southeast region); 5 144 322 individuals met the USPSTF 2013 criteria for LCS (2 090 636 female [40.6%]; 2 290 219 [44.5%] aged 61-70 years; 66 430 Asian or Indigenous [1.3%]; 491 527 Black [9.6%]; 2 073 836 *Pardo* [40.3%]; 2 512 529 [48.8%] White; 2 436 221 [47.4%] with an incomplete middle school education; and 2 577 300 [50.1%] living in the Southeast region), and 8 380 279 individuals met the USPSTF 2021 LCS criteria (3 507 760 female [41.9%]; 4 352 740 [51.9%] aged 50-60 years; 119 925 Asian or Indigenous [1.4%]; 839 171 Black [10.0%]; 3 330 497 *Pardo* [39.7%]; 4 090 687 [48.8%] White; 4 022 784 [48.0%] with an incomplete middle school education; and 4 162 070 [49.7%] living in the Southeast region). The number needed to screen to prevent 1 death was 177 individuals according to the USPSTF 2013 criteria and 242 individuals according to the USPSTF 2021 criteria. The YLG was 23 for all ever-smokers, 19 for the USPSTF 2013 criteria, and 21 for the USPSTF 2021 criteria. Being Black, having less than a high school education, and living in the North and Northeast regions were associated with increased 5-year risk of LC death.

**Conclusions and Relevance:**

In this comparative effectiveness study, USPSTF 2021 criteria were better than USPSTF 2013 in reducing disparities in LC death rates. Nonetheless, the risk of LC death remained unequal, and these results underscore the importance of identifying an appropriate approach for high-risk populations for LCS, considering the local epidemiological context.

## Key Points

**Question:**

Is the US Preventive Services Task Force (USPSTF) 2021 recommendation more effective in reducing sociodemographic disparities in lung cancer death risk (LCDR) compared with the USPSTF 2013 criteria?

**Findings:**

In this comparative effectiveness research study of a representative sample of the Brazilian population (>27 million individuals), use of the USPSTF 2021 criteria was associated with decreased disparity in LCDR between different demographic groups compared with the USPSTF 2013 criteria. Nevertheless, Black individuals continued to have the highest risk compared with other racial and ethnic groups, and individuals with low education levels and residing in low-resource areas also had higher LCDR than individuals with higher education and more resources.

**Meaning:**

These findings suggest that applying USPSTF 2021 guidelines rather than 2013 guidelines was associated with a reduction in disparities in LCDR, underscoring the importance of identifying an appropriate approach for high-risk populations for lung cancer screening, considering the local epidemiological context.

## Teaser

This comparative effectiveness research study examines whether the US Preventive Services Task Force (USPSTF) 2021 recommendations are more effective than the USPSTF 2013 criteria in reducing sociodemographic disparities in lung cancer death risk in Brazil.

## Introduction

Lung cancer (LC) is a major public health problem and was the second most frequent cancer and the most frequent cause of cancer-related mortality worldwide in 2020.^1^ In Brazil, LC was the deadliest cancer in 2020,^1^ and it is estimated that LC will be the second most frequent cancer (not accounting for breast and prostate cancers) from 2023 to 2025.^2^

The worldwide 5-year overall survival rate for LC is 18% (15% for men and 21% for women),^3^ with the prognosis depending on the stage at diagnosis. For those with localized disease, the 5-year survival rate is 59%.^4^ In Brazil, only 16% of patients receive a diagnosis at an early stage.^3^ In countries with LC screening (LCS) policies, early-stage diagnosis reaches 30% in the US^4^ and 34% in China,^5^ and its widespread adoption could potentially increase early-stage diagnosis by up to 70%.^6,7^

Therefore, implementing LCS in Brazil could improve early-stage diagnosis and, consequently, survival.^3,8^ The cost-effectiveness of LCS strategies depends on focusing on high-risk populations, such as individuals older than 50 years, smokers, and former smokers.^9,10^ In addition, the literature shows that LC disproportionately affects individuals from minoritized racial groups^11,12,13^ and socioeconomically disadvantaged groups.^4,14,15^

In 2011, the US National Lung Screening Trial (NLST)^6^ reported that screening with low-dose computed tomography (LDCT) reduces LC mortality by up to 20% among current and former smokers.^6,16,17^ The Nederlands Leuvens Longkanker Screenings Onderzoek (NELSON) study showed a 24% reduction in mortality in men (primary analysis) and 33% in women with 4 screenings over 5.5 years.^18^

In 2013, the US Preventive Services Task Force (USPSTF) recommended LDCT for ever-smokers aged 55 to 80 years with a history of 30 or more pack-years and less than 15 years since cessation.^19^ In July 2020, considering new evidence, the USPSTF expanded the criteria to individuals aged 50 to 80 years with 20 pack-years while maintaining the 15 years since cessation.^4,18,19^

In Brazil, LCS is feasible among ever-smokers with 30 pack-years and has a detection rate similar to that of the NLST, with most cancers diagnosed at stages IA and IB.^20,21^ In 2021, Miranda-Filho et al^22^ compared potential LCS strategies in Brazil using a telephone survey database covering 15 Brazilian capitals and estimated that the pack-year strategy can potentially identify 57.1% of preventable LC deaths if 21.8% of ever-smokers are screened.

Despite current efforts, there is still no consensus on how best to define the target population for LCS.^23,24,25^ The eligibility criteria for LCS determine its impact and efficiency. Establishing the requirements that best suit each country must consider the population of current and former smokers and their unique demographic structure related to age and smoking prevalence. Therefore, national modeling studies are essential to understand the possible effects of different strategies. In this study, we compared the number of individuals eligible for screening, 5-year preventable LC deaths, and years of life gained (YLG) if LC death is averted due to screening in Brazil, considering 3 different eligibility strategies: (1) ever-smokers aged 50 to 80 years, (2) those eligible for LCS according to USPSTF 2013 criteria, and (3) those eligible for LCS according to USPSTF 2021 criteria, stratifying the analysis by sociodemographic characteristics.

## Methods

### Study Design, Settings, and Data Source

This comparative effectiveness research study used the Brazilian National Household Survey (BNHS) from 2019 to estimate the population of current and former smokers in Brazil, their smoking history, and their risk characteristics for developing LC. We consider the data from adult residents (aged ≥18 years) who answered the specific questionnaire on health status, lifestyle, and chronic diseases, totaling 88 943 individuals.^26^

The BNHS 2019 sample for each eligibility strategy was 13 610 ever-smokers aged 50 to 80 years, 3009 individuals who met the USPSTF 2013 criteria for LCS, and 4843 individuals who met the USPSTF 2021 criteria for LCS. We estimated the number and percentage of current and former smokers eligible for LCS by LDCT in the 3 strategies, extrapolating the BNHS data to the Brazilian population by geographic location, sex, age, race (self-declared; Asian or Indigenous, Black, *Pardo *[which translates from Portuguese as “brown” or “mixed” and is used to denote individuals with predominantly Black and also mixed ancestry, including European, African, and Indigenous backgrounds],^27^^,^^28^ and White), and educational level. The inclusion of race in this analysis is a crucial aspect because of previously established connections between ethnic group, socioeconomic status, and the development of LC. Disparities in health outcomes have been documented across different racial and ethnic populations, uncovering patterns that extend beyond genetic factors to encompass social determinants of health. Certain racial and ethnic groups may experience distinct environmental exposures, access to health care, and lifestyle factors that contribute to variations in LC risk.

This study used secondary data available in the public domain, so it was exempt from ethical review and the need for informed consent according to the Brazilian National Ethics Committee and National Health Council Resolutions 466/2012^29^ and 510/2016.^30^ We followed the Strengthening the Reporting of Observational Studies in Epidemiology (STROBE) reporting guidelines for this comparative effectiveness study.^31^

### Estimation of Outcomes of Lung Cancer Death Risk Assessment Tool and Lung Cancer Risk Assessment Tool

We modified the full version of the Lung Cancer Death Risk Assessment Tool (LCDRAT) and the Lung Cancer Risk Assessment Tool (LCRAT) to estimate 5-year lung cancer deaths and 5-year risk of developing lung cancer in the absence of screening in the Brazilian population.^32^ We did not include the following components: family history of LC, liver disease in the last year, and coronary heart disease as a discrete variable. We adjusted the education and race categories to have equivalence to the BNHS 2019.

LCDRAT calculates the risk of LC death in the absence of screening, and LCRAT estimates the incidence of LC within 5 years. These models were developed using data from the Prostate, Lung, Colorectal, and Ovarian (PLCO) Cancer Screening Trial^33^ and have also been validated in US cohorts.^34^ In Brazil, a simplified version was applied to ever-smokers aged 55 to 79 years residing in 15 large cities in 3 regions of the country.^22^

We calculated the following rates per 1000: number of LC deaths, screening reduced LC deaths, number of LCs diagnosed, screening increased LC diagnosis, and false-positive lung CT scans. We also estimated the population eligible for screening and the mean age among screening-eligible individuals. Applying the previously mentioned rates, we obtained the number of LC deaths in the absence of screening, preventable LC deaths, the number needed to screen (NNS) to avoid 1 LC death, the 5-year LC death risk, LC cases in the absence of screening, screening increases LC diagnosis, the NNS to diagnose 1 LC case, and the 5-year LC risk. In addition, we calculated the YLG if LC is detected early due to screening and the YLG if LC death is averted due to screening.

### Comparing the Outcomes of LCS Eligibility Criteria

We compared the following LCS strategies: (1) ever-smokers aged 50 to 80 years, (2) USPSTF 2013 criteria (ie, adults aged 55 to 80 years who have a 30-pack-year smoking history and currently smoke or have quit in the past 15 years),^19^ and (3) USPSTF 2021 criteria (ie, adults aged 50 to 80 years who have a 20-pack-year smoking history and currently smoke or have quit in the past 15 years).^19^ The primary outcomes were population eligible for screening, the NNS to prevent 1 LC death, the 5-year LC death risk, and the YLG if LC death is averted due to screening; the analysis was performed considering possible outcomes for vulnerable populations, including geographic location, age, sex, race, and educational level as strata.

To our knowledge, these models have never been evaluated in the Brazilian population to estimate accuracy or calibration using sociodemographic variables. Nevertheless, there is strong evidence in the literature^35,36,37^ that in Brazil, minoritized groups are a more vulnerable population than the dominant groups, comparable to what is found in the US.

### Statistical Analysis

Data analysis was performed from February to May 2023. We applied the LCDRAT and LCRAT risk calculators available online^32^; their respective statistical methods are described elsewhere.^32^ Data were organized using Excel (Microsoft), and statistical tests were performed using SPSS statistical software version 28 (IBM). Data sets and specific output from the models are available in an online data repository.^38^ We performed 2-sided analysis of variance test to verify the differences in means at significance level of *P* < .05.

The number of pack-years was missing for 2102 of 35 236 (6%) current or past smokers included in the BNHS 2019. Data on race were missing for 70 smokers (0.002%), and all other variables did not have missing data. All missing records were excluded from the analysis.

## Results

The Brazilian population eligible for LCS, estimated on the basis of BNHS 2019 data, was 27 280 920 when considering all current and former smokers aged 50 to 80 years, of whom 13 387 552 (49.1%) were female, 13 249 531 (48.6%) were aged 50 to 60 years old, 394 994 were Asian or Indigenous (1.4%), 3 111 676 were Black (11.4%), 10 942 640 were *Pardo* (40.1%), 12 830 904 (47.0%) were White, 12 428 536 (45.6%) had an incomplete middle school education, and 12 860 132 (47.1%) lived in the Southeast region. When applying USPSTF 2013 criteria, 5 144 322 individuals were eligible for LCS: 2 090 636 (40.6%) were female, 2 290 219 (44.5%) were aged 61 to 70 years old, 66 430 were Asian or Indigenous (1.3%), 491 527 were Black (9.6%), 2 073 836 were *Pardo* (40.3%), 2 512 529 (48.8%) were White, 2 436 221 (47.4%) had an incomplete middle school education, and 2 577 300 (50.1%) lived in the Southeast region. Finally, when using USPSTF 2021 criteria, 8 380 279 individuals were eligible for LCS: 3 507 760 (41.9%) were female, 4 352 740 (51.9%) were aged 50 to 60 years, 119 925 were Asian or Indigenous (1.4%), 839 171 were Black (10.0%), 3 330 497 were *Pardo* (39.7%), 4 090 687 (48.8%) were White, 4 022 784 (48.0%) had an incomplete middle school education, and 4 162 070 (49.7%) lived in the Southeast region (Table 1). The 95% CIs are presented in eTables 1, 2, and 3 in Supplement 1.

**Table 1.** Estimated Number and Percentage of Current and Former Smokers Eligible for Low-Dose Computed Tomography Lung Cancer Screening, Brazilian National Health Survey 2019

Characteristic	Participants, No. (%)								
	All ever-smokers aged 50-80 y			USPSTF 2013 criteria^a^			USPSTF 2021 criteria^b^		
	Current smokers^c^	Former smokers^c^	Total^d^	Current smokers^c^	Former smokers^c^	Total^d^	Current smokers^c^	Former smokers^c^	Total^d^
Total	7762 862 (28.5)	19 518 057 (71.5)	27 280 920 (100.0)	2 992 053 (58.2)	2 152 269 (41.8)	5 144 322 (100.0)	4 944 641 (59.0)	3 435 638 (41.0)	8 380 279 (100.0)
Sex									
Female	3 592 661 (26.8)	9 794 891 (73.2)	13 387 552 (49.1)	1 161 642 (55.6)	928 995 (44.4)	2 090 636 (40.6)	2 054 256 (58.6)	1 453 504 (41.4)	3 507 760 (41.9)
Male	4 170 201 (30.0)	9 723 167 (70.0)	13 893 368 (50.9)	1 830 411 (59.9)	1 223 274 (40.1)	3 053 686 (59.4)	2 890 385 (59.3)	1 982 134 (40.7)	4 872 519 (58.1)
Age, y									
50-60	4 603 500 (34.7)	8 646 030 (65.3)	13 249 531 (48.6)	1 279 300 (66.2)	653 295 (33.8)	1 932 595 (37.6)	2 736 254 (62.9)	1 616 486 (37.1)	4 352 740 (51.9)
61-70	2 386 683 (25.8)	6 863 457 (74.2)	9 250 139 (33.9)	1 302 208 (56.9)	988 011 (43.1)	2 290 219 (44.5)	1 709 796 (58.5)	1 213 194 (41.5)	2 922 991 (34.9)
71-80	772 679 (16.2)	4 008 570 (83.8)	4 781 250 (17.5)	410 545 (44.6)	510 963 (55.4)	921 508 (17.9)	498 591 (45.1)	605 958 (54.9)	1 104 549 (13.2)
Race									
Black	885 641 (28.5)	2 226 035 (71.5)	3 111 676 (11.4)	293 662 (59.7)	197 864 (40.3)	491 527 (9.6)	509 061 (60.7)	330 110 (39.3)	839 171 (10.0)
*Pardo*^e^	3 265 194 (29.8)	7 677 445 (70.2)	10 942 640 (40.1)	1 265 478 (61.0)	808 358 (39.0)	2 073 836 (40.3)	1 993 796 (59.9)	1 336 700 (40.1)	3 330 497 (39.7)
White	3 489 611 (27.2)	9 341 292 (72.8)	12 830 904 (47.0)	1 384 521 (55.1)	1 128 008 (44.9)	2 512 529 (48.8)	2 358 193 (57.6)	1 732 494 (42.4)	4 090 687 (48.8)
Asian or Indigenous	122 416 (31.0)	272 579 (69.0)	394 994 (1.4)	48 392 (72.8)	18 038 (27.2)	66 430 (1.3)	83 592 (69.7)	36 333 (30.3)	119 925 (1.4)
Education level									
None	1 081 330 (31.4)	2 363 701 (68.6)	3 445 030 (12.6)	522 639 (71.0)	213 687 (29.0)	736 326 (14.3)	715 303 (69.0)	321 787 (31.0)	1 037 090 (12.4)
Incomplete middle school	3 677 812 (29.6)	8 750 725 (70.4)	12 428 536 (45.6)	1 392 146 (57.1)	1 044 074 (42.9)	2 436 221 (47.4)	2 399 973 (59.7)	1 622 812 (40.3)	4 022 784 (48.0)
Incomplete high school	838 796 (26.8)	2 290 641 (73.2)	3 129 437 (11.5)	300 241 (53.2)	264 418 (46.8)	564 659 (11.0)	511 136 (51.7)	476 959 (48.3)	988 095 (11.8)
High school	1 261 210 (27.2)	3 368 363 (72.8)	4 629 572 (17.0)	489 694 (56.3)	380 050 (43.7)	869 745 (16.9)	806 522 (58.2)	580 121 (41.8)	1 386 643 (16.5)
Incomplete college	174 071 (31.4)	379 977 (68.6)	554 049 (2.0)	64 481 (51.8)	60 115 (48.2)	124 596 (2.4)	105 983 (56.9)	80 289 (43.1)	186 272 (2.2)
College	729 644 (23.6)	2 364 651 (76.4)	3 094 295 (11.3)	222 850 (54.0)	189 925 (46.0)	412 775 (8.0)	405 725 (53.4)	353 669 (46.6)	759 394 (9.1)
Geographic region									
North	361 443 (26.6)	999 216 (73.4)	1 360 659 (5.0)	130 937 (62.1)	80 057 (37.9)	210 994 (4.1)	206 554 (60.6)	134 177 (39.4)	340 732 (4.1)
Northeast	1 734 124 (25.1)	5 170 768 (74.9)	6 904 892 (25.3)	667 127 (60.4)	437 630 (39.6)	1 104 757 (21.5)	1 087 001 (59.6)	736 412 (40.4)	1 823 413 (21.8)
Southeast	3 732 701 (29.0)	9 127 431 (71.0)	12 860 132 (47.1)	1 455 596 (56.5)	1 121 703 (43.5)	2 577 300 (50.1)	2 400 221 (57.7)	1 761 849 (42.3)	4 162 070 (49.7)
South	1 382 500 (31.7)	2 976 320 (68.3)	4 358 821 (16.0)	535 044 (57.9)	389 672 (42.1)	924 716 (18.0)	907 974 (60.4)	595 286 (39.6)	1 503 261 (17.9)
Midwest	552 093 (30.7)	1 244 323 (69.3)	1796 416 (6.6)	203 349 (62.3)	123 207 (37.7)	326 556 (6.3)	342 891 (62.3)	207 913 (37.7)	550 804 (6.6)

Abbreviation: USPSTF, US Preventive Services Task Force.

^a^ USPSTF 2013 criteria included adults aged 55 to 80 years who have a 30-pack-year smoking history and currently smoke or have quit within the past 15 years.

^b^ USPSTF 2021 criteria included adults aged 50 to 80 years who have a 20-pack-year smoking history and currently smoke or have quit within the past 15 years.

^c^ Percentages pertain to the row.

^d^ Percentages pertain to the column.

^e^ *Pardo* translates from Portuguese as “brown” or “mixed” and is used to denote individuals with predominantly Black and also mixed ancestry, including European, African, and Indigenous backgrounds.

Table 1 shows the estimated number and percentage of current and former smokers aged 50 to 80 years by sociodemographic characteristics. The percentage of tobacco use was higher in men than in women. The percentage of current smokers decreased with increasing age. Individuals from racial groups other than White and those with low education levels had higher rates of current smoking. The South and Southeast geographic regions had a higher percentage of current smokers.

The sociodemographic characteristics of the Brazilian populations eligible for screening were similar according to both the USPSTF 2013 and 2021 criteria (Table 1). In the 2013 USPSTF criteria, there were fewer female current smokers and a higher proportion of current smokers in the younger group compared with the 2021 criteria. It is important to note that the youngest group analyzed was different: 55 to 60 years in the USPSTF 2013 criteria vs 50 to 60 years in the USPSTF 2021 criteria.

The percentage of current smokers decreased with increasing age according to both sets of criteria. Regarding race, the percentage of eligible individuals in each group did not vary considerably considering the USPSTF 2013 and USPSTF 2021 criteria. Nevertheless, there was an increase in eligibility for current smokers with incomplete college degrees in the USPSTF 2021 criteria.

Table 2 shows the estimated outcomes of LCS under different eligibility strategies in Brazil. The 5-year LC death risk increased as the probability of meeting the measured end point increased; therefore, the most restrictive criterion, USPSTF 2013, would provide a higher chance of death from LC. We calculated the NNS needed to prevent 1 LC death on the basis of this mortality rate. According to the USPSTF 2013 criteria, we would need to screen 177 individuals to prevent 1 death, whereas following the USPSTF 2021 recommendation, the NNS would be 242. For the entire population, there would be 23 YLG if LC death was averted due to screening. The YLG would be 19 for the USPSTF 2013 criteria and 21 for the USPSTF 2021.

**Table 2.** Estimated Outcomes of Lung Cancer Screening Under Different Eligibility Strategies, Brazilian National Health Survey 2019

Model and outcomes	Mean (95% CI)			*P* value^c^
	All current and former smokers aged 50-80 y	USPSTF 2013 criteria^a^	USPSTF 2021 criteria^b^	
Lung Cancer Death Risk Assessment Tool				
Population eligible for screening, No. (%)	27 280 920 (100)	5 144 322 (19)	8 380 279 (31)	<.001
Age of screening-eligible individuals, y	62.2 (62.0-62.3)	64.2 (63.9-64.4)	61.7 (61.5-61.9)	<.001
False-positive computed tomography lung screens/1000	316.0 (315.0-317.0)	382.0 (381.0-384.0)	362.0 (361.0-364.0)	<.001
Lung cancer deaths/1000	10.4 (10.1-10.7)	27.7 (26.8-28.6)	20.3 (19.6-20.9)	<.001
Screening-reduced lung cancer deaths/1000	2.1 (2.1-2.2)	5.6 (5.5-5.8)	4.1 (4.0-4.3)	<.001
Lung cancer deaths in the absence of screening	283 482.0 (276 043.0-290 921.0)	142 296.2 (137 658.0-146 934.0)	169 927.3 (164 595.0-175 259.0)	<.001
Preventable lung cancer deaths eligible	57 830.3 (56 313.0-59 348.0)	29 028.4 (28 082.0-29 975.0)	34 665.2 (33 577.0-35 753.0)	<.001
NNS to prevent 1 lung cancer death	471.7 (460.0-484.0)	177.2 (172.0-183.0)	241.7 (234.0-250.0)	<.001
5-y Lung cancer death risk	1.00 (1.00-1.10)	2.80 (2.68-2.86)	2.00 (2.00-2.09)	<.001
Lung Cancer Risk Assessment Tool				
Percentage of preventable lung cancer deaths eligible	100.0	50.2	59.9	<.001
Patients with lung cancer diagnosed/1000	15.6 (15.2-15.9)	40.5 (39.2-41.7)	30.4 (29.6-31.4	<.001
Screening-related increase in lung cancer diagnosis/1000	1.9 (1.9-2.0)	5.0 (4.9-5.2)	3.8 (3.7-3.9)	<.001
Lung cancer cases in the absence of screening	424 268.0 (413 937.0-434 600.0)	208 138.0 (201 906.0-214 370.0)	255 104.0 (247 886.0-262 322.0)	<.001
Screening-related increase lung cancer diagnosis	52 609.0 (51 328.0-53 890.0)	25 809.0 (25 036.0-26 582.0)	31 633.0 (30 738.0-32 528.0)	<.001
NNS to diagnosis 1 lung cancer case	519.0 (506.0-532.0)	199.0 (194.0-205.0)	265.0 (258.0-273.0)	<.001
5-y Lung cancer risk	1.60 (1.50-1.60)	4.00 (4.00-4.20)	3.00 (3.00-3.10)	<.001
YLG				
YLG if lung cancer is found early due to screening	2.50 (2.46-2.48)	2.20 (2.10-2.20)	2.30 (2.20-2.30)	<.001
YLG if lung cancer death is averted due to screening	23.10 (23.00-23.20)	18.70 (18.50-18.80)	20.90 (20.80-21.10)	<.001

Abbreviations: NNS, number needed to screen; USPSTF, US Preventive Services Task Force; YLG, years of life gained.

^a^ USPSTF 2013 criteria included adults aged 55 to 80 years who have a 30-pack-year smoking history and currently smoke or have quit within the past 15 years.

^b^ USPSTF 2021 criteria included adults aged 50 to 80 years who have a 20-pack-year smoking history and currently smoke or have quit within the past 15 years.

^c^ *P* values were calculated with 2-sided analysis of variance.

The sociodemographic aspects of the 3 LCS strategies are shown in Table 3, Table 4, and Table 5; full data are shown in eTables 4, 5, and 6 in Supplement 1. Table 3 shows the population aged 50 to 80 years with any smoking history. The 5-year risk of LC death was significantly lower than that for those aged 50 to 60 years and with higher education. In the more restrictive criterion, USPSTF 2013, all risks are higher than 1. As for the USPSTF 2021 criteria, the risks were consistently higher than 1, except for the young group (aged 50-60 years).

**Table 3.** Estimated Outcomes of Lung Cancer Screening for Current and Former Smokers Aged 50-80 Years by Sociodemographic Characteristics, Brazilian National Health Survey 2019

Characteristic	All smokers			Lung Cancer Death Risk Assessment Tool		Lung Cancer Risk Assessment Tool		Years of life gained if lung cancer death is averted due to screening, mean (95% CI)
	Total population eligible for screening	Age among screening-eligible individuals, y, mean (95% CI)	False-positive CT lung screens/1000, mean (95% CI)	NNS to prevent 1 lung cancer death (95% CI)	5-y Lung cancer risk, mean (95% CI), %	NNS to diagnosis 1 lung cancer case, mean (95% CI)	5-y Lung cancer risk, mean (95% CI), %	
Geographic region								
North	1 360 659	61.8 (61.4-62.2)	315 (312-318)	442 (411-478)	1.1 (1.0-1.2)	499 (466-536)	1.6 (1.5-1.7)	23.5 (23.1-23.8)
Northeast	6 904 892	62.3 (62.0-62.5)	316 (315-318)	450 (430-472)	1.1 (1.0-1.1)	501 (480-523)	1.6 (1.5-1.7)	23.2 (22.9-23.4)
Southeast	12 860 132	62.4 (62.2-62.7)	316 (314-318)	499 (475-525)	1.0 (0.9-1.0)	542 (518-568)	1.5 (1.4-1.6)	22.9 (22.7-23.1)
South	4 358 821	62.2 (61.9-62.6)	320 (318-322)	469 (441-500)	1.0 (1.0-1.1)	503 (476-534)	1.6 (1.5-1.7)	22.7 (22.4-23.0)
Midwest	1 796 416	61.6 (61.2-62.0)	311 (309-314)	525 (487-570)	0.9 (0.9-1.0)	571 (532-617)	1.4 (1.3-1.5)	23.7 (23.3-24.0)
Age group, y								
50-60	13 249 531	55.2 (55.2-55.3)	291 (289-292)	1125 (1091-1161)	0.4 (0.4-0.5)	1068 (1039-1098)	0.8 (0.7-0.8)	28.7 (28.6-28.8)
61-70	9 250 139	65.1 (65-65.2)	329 (327-330)	414 (401-429)	1.2 (1.1-1.2)	452 (438-467)	1.8 (1.7-1.8)	20.4 (20.3-20.6)
71-80	4 781 250	74.8 (74.7-74.9)	358 (356-360)	207 (198-218)	2.4 (2.3-2.5)	249 (238-262)	3.2 (3.1-3.4)	13.5 (13.4-13.6)
Sex								
Male	13 893 368	62.6 (62.4-62.8)	323 (322-325)	393 (380-407)	1.2 (1.2-1.3)	448 (435-463)	1.80 (1.74-1.86)	21.6 (21.5-21.8)
Female	13 387 552	61.7 (61.5-61.9)	307 (306-309)	621 (597-647)	0.8 (0.76-0.82)	639 (616-663)	1.3 (1.2-1.3)	24.9 (24.7-25.1)
Race								
White	12 830 904	62.7 (62.5-62.9)	321 (319-322)	461 (443-480)	1.1 (1.0-1.1)	484 (467-503)	1.7 (1.6-1.7)	22.5 (22.4-22.7)
Black	3 111 676	61.9 (61.5-62.3)	338 (335-341)	280 (262-301)	1.7 (1.6-1.9)	340 (319-363)	2.4 (2.2-2.5)	21.1 (20.8-21.4)
*Pardo*^a^	10 942 640	61.8 (61.6-62.0)	306 (305-308)	582 (561-605)	0.8 (0.8-0.9)	642 (620-665)	1.3 (1.2-1.3)	24.1 (23.9-24.3)
Asian or Indigenous	394 994	62.8 (61.7-64.0)	304 (296-311)	676 (555-863)	0.7 (0.6-0.9)	687 (568-870)	1.2 (0.9-1.4)	25.0 (24.0-26.1)
Education level								
Incomplete high school	19 003 004	62.6 (62.5-62.8)	324 (322-325)	405 (394-418)	1.2 (1.2-1.3)	452 (440-465)	1.8 (1.7-1.8)	22.2 (22.1-22.4)
High school	4 629 572	60.3 (60.1-60.6)	301 (299-304)	707 (665-754)	0.7 (0.6-0.7)	746 (704-793)	1.1 (1.0-1.1)	25.1 (24.8-25.4)
Incomplete college	554 049	61.0 (60.1-61.9)	301 (295-307)	851 (728-1024)	0.6 (0.5-0.7)	833 (719-991)	1.0 (0.8-1.1)	25.0 (24.2-25.8)
College	3 094 295	62.0 (61.7-62.4)	290 (288-293)	975 (898-1066)	0.50 (0.46-0.55)	946 (878-1026)	0.9 (0.8-0.9)	25.7 (25.3-26.0)
Total	27 280 920	62.2 (62.0-62.3)	316 (315-317)	472 (460-484)	1.0 (1.01-1.07)	519 (506-532)	1.6 (1.5-1.6)	23.1 (23.0-23.2)

Abbreviations: CT, computed tomography; NNS, number needed to screen.

^a^ *Pardo* translates from Portuguese as “brown” or “mixed” and is used to denote individuals with predominantly Black and also mixed ancestry, including European, African, and Indigenous backgrounds

**Table 4.** Estimated Outcomes of Lung Cancer Screening Under US Preventive Services Task Force 2013 Eligibility Criteria by Sociodemographic Characteristics, Brazilian National Household Survey 2019^a^

Characteristic	All smokers			Lung Cancer Death Risk Assessment Tool		Lung Cancer Risk Assessment Tool		Years of life gained if lung cancer death is averted due to screening, mean (95% CI)
	Total population eligible for screening	Age among screening-eligible individuals, y, mean (95% CI)	False-positive CT lung screens/1000, mean (95% CI)	NNS to prevent 1 lung cancer death, mean (95% CI)	5-y Lung cancer risk, mean (95% CI), %	NNS to diagnosis 1 lung cancer case, mean (95% CI)	5-y Lung cancer risk, mean (95% CI), %	
Geographic region								
North	210 994	64.1 (63.4-64.7)	385 (381-389)	160 (147-175)	3.10 (2.80-3.34)	184 (170-200)	4.40 (4.03-4.74)	18.7 (18.2-19.2)
Northeast	1 104 757	64.8 (64.4-65.2)	386 (383-389)	163 (154-172)	3.00 (2.84-3.18)	186 (177-196)	4.30 (4.11-4.55)	18.2 (17.9-18.6)
Southeast	2 577 300	64.1 (63.7-64.6)	379 (376-382)	195 (183-209)	2.50 (2.35-2.68)	215 (203-229)	3.70 (3.52-3.97)	18.8 (18.4-19.1)
South	924 716	63.7 (63.1-64.2)	382 (379-386)	186 (173-202)	2.60 (2.43-2.84)	204 (191-220)	4.00 (3.67-4.23)	18.8 (18.3-19.3)
Midwest	326 556	63.4 (62.7-64.1)	377 (372-381)	199 (181-222)	2.50 (2.21-2.71)	221 (202-245)	3.60 (3.29-3.99)	19.3 (18.7-19.9)
Age group, y								
50-60	1 932 595	57.5 (57.4-57.6)	353 (351-355)	397 (382-414)	1.20 (1.18-1.28)	403 (390-418)	2.00 (1.93-2.07)	23.6 (23.4-23.8)
61-70	2 290 219	64.9 (64.8-65.1)	387 (385-388)	184 (178-191)	2.70 (2.57-2.76)	204 (198-211)	3.90 (3.82-4.07)	17.9 (17.7-18.0)
71-80	921 508	74.7 (74.5-75.0)	426 (424-429)	84 (80-88)	5.90 (5.58-6.13)	100 (95-105)	8.10 (7.72-8.46)	11.4 (11.2-11.7)
Sex								
Male	3 053 686	64.2 (63.9-64.5)	387 (385-389)	160 (154-167)	3.10 (2.93-3.18)	185 (178-192)	4.40 (4.21-4.52)	17.7 (17.5-17.9)
Female	2 090 636	64.1 (63.7-64.5)	374 (372-377)	217 (206-230)	2.30 (2.13-2.38)	232 (220-244)	3.50 (3.30-3.66)	20.3 (20.0-20.6)
Race								
White	2 512 529	64.3 (64.0-64.7)	387 (385-389)	173 (165-183)	2.80 (2.69-2.97)	186 (178-195)	4.30 (4.13-4.53)	18.4 (18.1-18.7)
Black	491 527	63.9 (63.2-64.6)	406 (402-410)	108 (100-117)	4.50 (4.18-4.91)	133 (124-144)	6.00 (5.59-6.50)	16.7 (16.1-17.2)
*Pardo*^b^	2 073 836	64.1 (63.8-64.4)	373 (371-375)	216 (207-226)	2.30 (2.16-2.37)	245 (235-256)	3.30 (3.16-3.43)	19.4 (19.1-19.6)
Asian or Indigenous	66 430	64.8 (63.0-66.7)	374 (361-387)	229 (177-324)	2.10 (1.51-2.77)	232 (181-321)	3.50 (2.51-4.46)	20.8 (19.1-22.4)
Education level								
Incomplete high school	3 737 206	64.4 (64.2-64.7)	387 (385-389)	162 (156-168)	3.00 (2.92-3.14)	184 (178-190)	4.40 (4.23-4.52)	18.1 (17.9-18.4)
High school	869 745	62.9 (62.3-63.5)	370 (366-374)	243 (224-264)	2.00 (1.86-2.18)	263 (244-284)	3.10 (2.84-3.31)	19.9 (19.4-20.4)
Incomplete college	124 596	63.1 (61.5-64.7)	359 (349-370)	325 (263-426)	1.50 (1.15-1.86)	331 (270-428)	2.40 (1.88-2.98)	21.3 (20-22.7)
College	412 775	64.0 (63.2-64.8)	360 (355-365)	313 (278-358)	1.60 (1.37-1.77)	308 (277-348)	2.60 (2.32-2.91)	21.1
Total	5 144 322	64.2 (63.9-64.4)	382 (381-384)	177 (172-183)	2.80 (2.68-2.86)	199 (194-205)	4.00 (3.92-4.17)	18.7 (18.5-18.8)

Abbreviations: CT, computed tomography; NNS, number needed to screen.

^a^ The 2013 criteria include adults aged 55 to 80 years who have a 30-pack-year smoking history and currently smoke or have quit within the past 15 years.

^b^ *Pardo* translates from Portuguese as “brown” or “mixed” and is used to denote individuals with predominantly Black and also mixed ancestry, including European, African, and Indigenous backgrounds

**Table 5.** Estimated Outcomes of Lung Cancer Screening Under US Preventive Services Task Force 2021 Eligibility Criteria by Sociodemographic Characteristics, Brazilian National Household Survey 2019^a^

Characteristic	All smokers			Lung Cancer Death Risk Assessment Tool		Lung Cancer Risk Assessment Tool		Years of life gained if lung cancer death is averted due to screening, mean (95% CI)
	Total population eligible for screening	Age among screening-eligible individuals, y, mean (95% CI)	False-positive CT lung screens/1000, mean (95% CI)	NNS to prevent 1 lung cancer death, mean (95% CI)	5-y Lung cancer risk, mean (95% CI), %	NNS to diagnosis 1 lung cancer case, mean (95% CI)	5-y Lung cancer risk, mean (95% CI), %	
Geographic region								
North	210 994	61.5 (61.0-62.1)	362 (358-366)	220 (202-241)	2.2 (2.0-2.4)	248 (230-270)	3.2 (3.0-3.5)	21.2 (20.6-21.7)
Northeast	1 104 757	62.0 (61.6-62.4)	364 (361-366)	226 (215-239)	2.2 (2.1-2.3)	252 (241-265)	3.2 (3.0-3.3)	20.8 (20.5-21.1)
Southeast	2 577 300	61.9 (61.5-62.3)	362 (359-364)	260 (245-277)	1.9 (1.8-2.0)	280 (265-296)	2.9 (2.7-3.0)	20.8 (20.4-21.1)
South	924 716	61.4 (60.9-61.9)	363 (360-366)	251 (234-272)	1.9 (1.8-2.1)	268 (251-287)	3.0 (2.8-3.2)	20.9 (20.4-21.3)
Midwest	326 556	60.8 (60.2-61.5)	357 (353-361)	270 (246-299)	1.8 (1.6-2.0)	292 (268-321)	2.8 (2.5-3.0)	21.6 (21.0-22.1)
Age group, y								
50-60	4 352 740	55.3 (55.2-55.4)	332 (331-334)	580 (561-601)	0.8 (0.8-0.9)	564 (548-581)	1.4 (1.4-1.5)	25.9 (25.8-26.1)
61-70	2 922 991	64.9 (64.8-65.1)	379 (378-381)	210 (203-218)	2.3 (2.3-2.4)	230 (223-238)	3.5 (3.4-3.6)	18.1 (17.9-18.3)
71-80	1 104 549	74.7 (74.5-74.9)	418 (416-421)	95 (91-100)	5.2 (4.9-5.4)	112 (107-117)	7.2 (6.9-7.5)	11.7 (11.5-11.9)
Sex								
Male	4 872 519	61.8 (61.5-62.1)	367 (366-369)	214 (206-223)	2.3 (2.2-2.4)	241 (233-250)	3.3 (3.2-3.5)	19.9 (19.7-20.1)
Female	3 507 760	61.6 (61.3-61.9)	354 (352-356)	303 (289-319)	1.6 (1.5-1.7)	313 (299-328)	2.6 (2.5-2.7)	22.6 (22.3-22.9)
Race								
White	4 090 687	62.0 (61.7-62.4)	368 (366-370)	232 (222-244)	2.1 (2.0-2.2)	243 (233-254)	3.3 (3.2-3.5)	20.4 (20.1-20.7)
Black	839 171	61.5 (60.9-62.1)	384 (381-388)	149 (138-161)	3.3 (3.0-3.6)	179 (167-194)	4.5 (4.2-4.8)	18.8 (18.3-19.3)
*Pardo*^b^	3 330 497	61.5 (61.2-61.8)	351 (349-353)	300 (287-314)	1.6 (1.6-1.7)	330 (318-344)	2.4 (2.3-2.5)	21.9 (21.6-22.2)
Asian or Indigenous	119 925	62.0 (60.1-63.9)	355 (344-366)	315 (245-440)	1.6 (1.1-2.0)	311 (246-423)	2.6 (1.9-3.3)	23.4 (21.7-25)
Education level								
Incomplete high school	6 047 970	62.0 (61.7-62.2)	367 (365-368)	220 (212-227)	2.2 (2.2-2.3)	244 (236-252)	3.3 (3.2-3.4)	20.4 (20.2-20.6)
High school	1 386 643	60.3 (59.8-60.8)	350 (346-353)	335 (311-363)	1.5 (1.4-1.6)	352 (329-379)	2.3 (2.1-2.5)	22.4 (21.9-22.9)
Incomplete college	186 272	60.4 (58.9-61.9)	342 (333-352)	438 (360-559)	1.1 (0.9-1.4)	430 (358-537)	1.9 (1.5-2.3)	23.4 (22.1-24.7)
College	759 394	61.9 (61.2-62.6)	340 (336-345)	429 (383-487)	1.1 (1.0-1.3)	414 (375-462)	1.9 (1.8-2.2)	23.2 (22.5-23.9)
Total	8 380 279	61.7 (61.5-61.9)	362 (361-364)	242 (234-250)	2.0 (2.0-2.1)	265 (258-273)	3.0 (3.0-3.1)	20.9 (20.8-21.1)

Abbreviations: CT, computed tomography; NNS, number needed to screen.

^a^ The 2021 criteria included adults aged 50 to 80 years who have a 20-pack-year smoking history and currently smoke or have quit within the past 15 years.

^b^ *Pardo* translates from Portuguese as “brown” or “mixed” and is used to denote individuals with predominantly Black and also mixed ancestry, including European, African, and Indigenous backgrounds

The 5-year LC death risk by race showed large variability, with a higher risk for Black individuals. The USPSTF 2013 had the most significant disparity between races, ranging from 2.14 for Asian or Indigenous people to 4.54 for Black people. In the USPSTF 2021, the gap narrowed in risk between races. Having less than a high school education was associated with increased 5-year risk of LC death, with high variability for the USPSTF 2013. This risk was also higher for individuals in the North and Northeast regions than for the other areas of the country.

When analyzing the YLG if LC death was averted due to screening, among sociodemographic characteristics, Black people had the largest gap with eligible population in each strategy, with a difference of 2 YLG. Independent of eligibility strategy, the YLG was similar across geographic regions, with a maximum 1-year difference. Conversely, sex showed a difference of 2 to 3 years, with women having a higher YLG than men. As might be expected, individuals with lower levels of education had fewer YLG, 3 to 4 years less than those with a college degree or higher.

## Discussion

The findings of this comparative effectiveness research study suggest that LDCT for LCS, independently of the LCS strategy adopted, can reduce the LC mortality rate in Brazil. It could potentially be a public policy to reduce the burden of LC in the country by broadening the focus of current health policy beyond prevention to early-stage LC diagnosis, which has been proven to increase survival, reduce mortality rates, and increase the YLG. Implementing the LCS program in Brazil will lead the country to a more up-to-date approach that has been proven to reduce the burden of cancer worldwide.

One of the main barriers to the adoption of LCS in Brazil is the high rate of granulomatosis due to the high incidence rate of tuberculosis, which potentially increases the false-positive rate and, consequently, the NNS, which yields losses in effectiveness for LCS. Nevertheless, the current literature^39,40,41,42^ shows that LCS programs have reduced mortality and increased early-stage LC diagnosis in Asian countries with high tuberculosis incidence rates. In Brazil, 2 studies^20,21^ found false-positive rates similar to those of the NLST, despite its small sampling. Implementation studies^43,44^ have also shown that LCS increased the rates of early-stage LC diagnosis. These results show that adopting LCS would have similar benefits in a population with a high prevalence of granulomatous disease, because it does not increase the number of false-positive results with a high suspicion of LC, avoiding unnecessary biopsies and surgical procedures.

LDCT for the use of LCS has shown excellent results in the US and China. However, it is worth noting that early-stage diagnosis is affected by the adoption of the LDCT. Therefore, barriers to access to LDCT, such as cost, should be considered.^5,45^ Brazil has universal health care coverage through the public health system, so there is no out-of-pocket payment for individuals, and the adoption of LCS as a public policy has notable potential.

Although there is growing evidence that LCS is a vital strategy to reduce LC mortality, the best screening criteria are still debated. Thus, several aspects should be considered in designing and implementing LCS programs, such as equity in eligibility regarding sociodemographic characteristics, strategies for physicians and the population at risk, and cost-effectiveness.

Similar to the findings of a study by Aldrich et al,^46^ we observed that reducing the smoking pack-year eligibility criteria to a minimum 20-pack-year history was associated with a higher percentage of screening eligibility of Black vs White smokers at all ages. The USPSTF 2021, when applied to the Brazilian population, reduces the disparities in sex, race, and educational level due to the decrease in pack-years compared with the USPSTF 2013. Nonetheless, it does not eliminate them. These results agree with other findings in the literature.^15,23,24,25,46,47,48^

Alternatively, instead of a single approach to LCS eligibility applied uniformly across the country, multiple strategies should be evaluated. Future analysis should consider different perspectives, including race, education level, and pack-years. Twenty pack-years may be better for screening those from socioeconomically disadvantaged backgrounds. These adjustments in strategies by population characteristics would increase sensitivity for eligibility criteria, promoting greater accuracy in calculating the NNS to prevent deaths from LC.

On the other hand, an individual risk calculator with a risk threshold could also be an option for an umbrella global approach to select the eligible population. The UK Lung Screening pilot trial demonstrated the possibility of detecting LC early and delivering potentially curative treatment in more than 80% of cases, using a population-based questionnaire to identify high-risk individuals.^49,50^

Although implementing an individual risk calculator in a shared decision-making process may be advantageous compared with a general population-wide criterion, it presents some challenges. First, shared decision-making tools are not designed for low health literacy or culturally diverse populations. Furthermore, individuals who smoke tend to be less educated and are less likely to have a primary care practitioner, which reduces access to LCS. There are also implicit biases based on sex, race, ethnicity, and caregivers’ perception that negatively affect communication and patient-practitioner interactions. In Brazil, Black individuals report discrimination in health services, including fewer laboratory test requests.^51,52,53^ Moreover, smoking carries a stigma, and many smokers have a high level of nihilism and lack of knowledge about LCS and its benefits. In addition, patients’ distrust of the health care system and practitioners harms access to preventive care.^15^

There are specific challenges in the implementation of LCS in Brazil. Despite universal health care coverage, the distribution of health services, trained professionals, and the availability of CT scans are uneven across the country and are limited in some areas. There is substantial variability among Brazilian geographic regions regarding health outcomes, such as life expectancy, and risk factors, such as smoking prevalence, environmental exposure, health-seeking behavior, and sociodemographic and economic characteristics.^54,55^

Current international evidence shows the relevance of LCS in reducing LC mortality and increasing survival due to early detection.^7,41,56^ Future studies should consider cost-effectiveness analysis as a component to define which criterion or approach would be more appropriate for the Brazilian population.

### Limitations

This study has limitations that should be mentioned. First, the potential harms associated with LCS, such as surveillance scans, unnecessary invasive procedures, and overdiagnosis, were not estimated. Second, we modified the full version of the LCDRAT and the LCRAT. These models assumed the 20% reduction in LC mortality observed in NLST applies to all potentially eligible current and former smokers, and this has not yet been validated in Brazil.^32^ Third, we did not include family history of LC, liver disease in the last year, or coronary heart disease as a discrete variable because of their unavailability in the BNHS.

Fourth, the LCDRAT was not calibrated for the Brazilian population, which is different from the US population in ways that could affect the model’s performance, and this could be a substantial limitation. Fifth, it was not possible to use the national mortality database (Sistema de Informação de Mortalidade) to validate the number of deaths estimated, because it does not include smoking history. An alternative would be to link hospital-based cancer registries with information on smoking history and the mortality information system. Nevertheless, this method would have its own limitations because we would not be able to estimate the smoking history of individuals who died of cancer but were not registered in the hospital-based cancer registries.

## Conclusions

This comparative effectiveness research study found that the 2021 USPSTF criteria are more equitable than the 2013 criteria with regard to LCS guidelines. Nevertheless, sociodemographic disparities remain. This may suggest that the USPSTF 2021 LCS guidelines need adjustments to be applied to the Brazilian population. Evidence shows that risk estimation models may be more efficient in selecting ever-smokers for screening than pack-year guidelines and comparing with an eligibility risk threshold. In Brazil, where there are no national guidelines on LCS, it is highly relevant that future studies explore different aspects of LCS strategies, such as the use of individual risk calculators vs umbrella criteria as well as cost-effectiveness.

## Notes

Supplement 1. **eTable 1.** Estimated Number and Percentage of Current and Former Smokers Aged 50-80 Years Old by Sociodemographic Characteristics in Brazil, 2019 **eTable 2.** Estimated Number and Percentage of Current and Former Smokers Eligible for Low-Dose CT Lung Cancer Screening Under USPSTF-2013 Eligibility Criteria by Sociodemographic Characteristics in Brazil, 2019 **eTable 3.** Estimated Number and Percentage of Current and Former Smokers Eligible for Low-Dose CT Lung Cancer Screening Under USPSTF-2021 Eligibility Criteria by Sociodemographic Characteristics in Brazil, 2019 **eTable 4.** Estimated Outcomes of Lung Cancer Screening for Current and Former Smokers Aged 50–80 by Sociodemographic Characteristics in Brazil, 2019 **eTable 5.** Estimated Outcomes of Lung Cancer Screening Under USPSTF-2013^a^ Eligibility Criteria by Sociodemographic Characteristics in Brazil, 2019 **eTable 6.** Estimated Outcomes of Lung Cancer Screening Under USPSTF-2021^b^ Eligibility Criteria by Sociodemographic Characteristics in Brazil, 2019

Supplement 2.
Data Sharing Statement
