# Supplementary material for: Lung Cancer Screening in Brazil Comparing the 2013 and 2021 USPSTF Guidelines
Source: JAMA Netw Open. 2023 Dec 11;6(12):e2346994. doi: 10.1001/jamanetworkopen.2023.46994 (PMC10714246; doi:10.1001/jamanetworkopen.2023.46994)
Supplement: Supplement 2. — Data Sharing Statement [file jamanetwopen-e2346994-s002.pdf]

Data Sharing Statement

Emmerick. Lung Cancer Screening in Brazil Comparing the 2013 and 2021 USPSTF Guidelines. *JAMA Netw Open*. Published December 11, 2023.
doi:10.1001/jamanetworkopen.2023.46994

Data
Data available: Yes
Data types: Data (not involving human participants)
How to access data: The datasets and respective calculators are available at the Oswaldo Cruz Foundation (FIOCRUZ-Brazil) repository and can be accessed at the following link: https://www.arca.fiocruz.br/handle/icict/60895.
When available: With publication

Supporting Documents
Document types: Statistical/analytic code
How to access documents: Calculators are available at the Oswaldo Cruz Foundation (FIOCRUZ-Brazil) repository and can be accessed at the following link: https://www.arca.fiocruz.br/handle/icict/60895.
When available: With publication

Additional Information
Who can access the data: Anyone who wish to access the data.
Types of analyses: Any purpose.
Mechanisms of data availability: The data will be available in the repository.
